# Redundant Postsynaptic Functions of SynCAMs 1–3 during Synapse Formation

**DOI:** 10.3389/fnmol.2017.00024

**Published:** 2017-01-31

**Authors:** Daniel K. Fowler, James H. Peters, Carly Williams, Philip Washbourne

**Affiliations:** ^1^Department of Biology, Institute of Neuroscience, University of OregonEugene, OR, USA; ^2^Department of Integrative Physiology and Neuroscience, Washington State UniversityPullman, WA, USA

**Keywords:** redundancy, SynCAM, artificial miRNA, mosaic, hippocampus, synapse formation, knockdown, adhesion

## Abstract

Investigating the roles of synaptogenic adhesion molecules during synapse formation has proven challenging, often due to compensatory functions between additional family members. The synaptic cell adhesion molecules 1–3 (SynCAM1–3) are expressed both pre- and postsynaptically, share highly homologous domains and are synaptogenic when ectopically presented to neurons; yet their endogenous functions during synaptogenesis are unclear. Here we report that SynCAM1–3 are functionally redundant and collectively necessary for synapse formation in cultured hippocampal neurons. Only triple knockdown (KD) of SynCAM1–3 using highly efficient, chained artificial microRNAs (amiRNAs) reduced synapse density and increased synapse area. Electrophysiological recordings of quantal release events supported an increase in synapse size caused by SynCAM1–3 depletion. Furthermore, a combinatorial, mosaic lentiviral approach comparing wild type (WT) and SynCAM1–3 KD neurons in the same culture demonstrate that SynCAM1–3 set synapse number and size through postsynaptic mechanisms. The results demonstrate that the redundancy between SynCAM1–3 has concealed their synaptogenic function at the postsynaptic terminal.

## Introduction

Synapse formation is initiated by physical contact of adhesion molecules between axons and dendrites which then triggers recruitment of molecular complexes to the presynaptic active zone or the postsynaptic density (PSD) (Washbourne et al., 2004). Despite considerable advances, a comprehensive understanding of how adhesion molecules orchestrate synaptogenesis is far from complete. A major obstacle in studying synapse formation is the confound of functional redundancy between key synaptic proteins. Indeed, redundancy has been observed for the synaptogenic adhesion molecule families neurexins, neuroligins and calsyntenins, such that protein reduction of at least three family members is necessary to observe certain synaptic phenotypes (Varoqueaux et al., 2006; Shipman et al., 2011; Gokce and Südhof, 2013; Um et al., 2014). Importantly, compensatory functions within protein families may hide crucial synaptic roles when manipulating single genes in isolation.

Several observations suggest that the nectin-like synaptic cell adhesion molecule (SynCAM) family may also share redundant functions during synapse formation. SynCAMs were discovered in the central nervous system by their ability to induce synapse formation *in vitro* (Biederer et al., 2002). They have been linked to autism spectrum disorder (Zhiling et al., 2008; Casey et al., 2012), and have four members (SynCAM1–4) that form homo- or heterophilic interactions in the *trans* configuration across the synaptic cleft (Fogel et al., 2007). SynCAMs 1, 2 and 3 (SynCAM1–3) are localized to excitatory synapses with both pre- and postsynaptic distributions (Fogel et al., 2007; Stagi et al., 2010; Shu et al., 2011; Cheadle and Biederer, 2012; Loh et al., 2016). The short intracellular regions of SynCAM1–3, requisite for synaptogenic activity (Biederer et al., 2002; Sara et al., 2005), contain remarkably conserved binding motifs between family members and likely interact with the same proteins during development (Biederer, 2006). While the roles of SynCAMs during synapse formation are emerging, the extent of overlap in SynCAM signaling remains unexplored.

Here we use single and chained artificial microRNAs (amiRNAs) to knock down SynCAM1–3 in cultured rat hippocampal neurons to examine functional redundancy between these gene family members. Knockdown (KD) of any single, or two SynCAMs does not affect excitatory synapse formation; rather triple KD of SynCAM1–3 shows they are necessary for and compensate to set synapse density and limit synapse size. We further investigate these phenotypes using a novel method that generates a traceable mosaic of KD and wild type (WT) cells on the same coverslip. Crucially, comparisons between these conditions allows for the differentiation of pre- and postsynaptic effects. Using this method we find that SynCAM1–3’s influence on synapse number and size are through postsynaptic mechanisms. Electrophysiological recordings confirm postsynaptic effects of SynCAM1–3 KD with broadened event peaks of miniature excitatory postsynaptic currents (mEPSCs) consistent with an increase in synapse size. These results suggest that a postsynaptic mechanism for SynCAM1–3 in synaptogenesis has been heretofore concealed by overlapping functions of the gene family members.

## Materials and Methods

### Cloning

The design and generation of amiRNAs targeting SynCAM1–3 and Scrambled1–3 amiRNAs, a MultiSite Gateway (Invitrogen) middle entry vector with an intronically-expressed enhanced synthetic inhibitory BIC/miR-155 RNA cloning cassette (pME-eSIBR), and insertion and chaining of amiRNAs in the cassette were described previously (Fowler et al., 2016b). SynCAM1–3 (*cadm1–3*) guide strand amiRNA targeting sequences used were *cadm1.1358*:5′-UUGAUUAUAGCUGUGUCUGCGU-3′, *cadm2.87*:5′-UUCAACAACCGUGACAUUCUGA-3′, and *cadm3.387*:5′-AUAACCAGUGAUUAUGGGUUUC-3′. Scrambled control guide strand sequences used were scrambled1: 5′-AUUCUAAUACUACGUUCCGCAU-3′, scrambled2: 5′-ACAACUUGUAUAUCGCGCAACU-3′ and scrambled3: 5′-GAUCUUAUACUCGUGAUUGAGA-3′. Gateway LR recombination reactions of pME-eSIBR vectors with single, double, or triple amiRNAs with a 5′ entry vector containing a minimal CMV (mCMV) promoter (p5E-CMVmin) and 3′ entry vector with a nlsGFP tag (p3E-nlsGFP no-pA) into a third-generation lentiviral destination vector (pEpic_Lite) were performed to create SynCAM single, double and triple KD vectors or the control Scrambled1–3 amiRNA vector were described previously (Fowler et al., 2016a). To make the memGFP-only expressing vector, a middle entry vector containing GFP with a C-terminal human H-RAS palmitoylation signal for membrane targeting (Kwan et al., 2007) was used in a MultiSite Gateway LR reaction with p5E-CMVmin, a 3′ entry vector with a hemagglutinin epitope tag (p3E-HA no-pA), and pEpic_Lite (Fowler et al., 2016a). The HA epitope is not expressed because the memGFP sequence used contains a stop codon and was used as a “filler” sequence to allow LR recombination.

### Lentivirus Production and Titration

2.5 × 10^6^ HEK293T cells (ATCC^®^ CRL-3216) were plated per 10-cm tissue culture dishes in 10 ml of DMEM (Invitrogen), 10%FCS (Atlanta Biologicals), 25 units/ml penicillin and 25 μg/ml streptomycin (Sigma). Approximately twenty-four hours after plating, cells were transiently transfected using ProFection (Promega) calcium phosphate transfection reagents with 20 μg pEpic_Lite lentiviral vectors and packaging vectors (10 μg pMDL g/p RRE, 5 μg pRSV-Rev, 6 μg pVSV-G; Dull et al., 1998). Six to Eight hours later, media was replaced with 6 ml/plate of fresh medium. Medium was collected 48–72 h after transfection and centrifuged at 3000× g for 5 min at room temperature (RT). Supernatant was passed through a 0.45 μm syringe filter and virus was concentrated by centrifugation on a 150,000 MWCO column (Pierce). Twenty thousand HEK293T cells were plated per well of a 12-well plate and transduced with serial dilutions of concentrated lentivirus. Four to five days after transduction, titers were calculated by flow cytometry on an Attune^®^ acoustic focusing cytometer (Applied Biosystems) for GFP+ cells. Infectious lentiviral particles/μl was calculated from viral dilutions where cells were transduced in the linear range (5%–20% GFP+ cells).

### Vertebrate Animals

Studies using rats were carried out in strict accordance with the recommendations in the Guide for the Care and Use of Laboratory Animals of the National Institutes of Health. The protocols were approved by the University of Oregon and Washington State University Institutional Animal Care and Use Committee (Permit Numbers: #13-19 and #04787, respectively). Rats were anesthetized with isoflurane prior to sacrifice and culturing of neurons. Rats were housed with a 12/12 light/dark cycle according to standard protocols in the University of Oregon Animal Care Facility and Washington State University Veterinary and Biomedical Research Vivarium. Sprague-Dawley rats were obtained from Envigo.

### Primary Neuron Culture

Hippocampal cultures were prepared from embryonic day 19 Sprague-Dawley rat pups as described (Brewer et al., 1993), with minimal modifications. For single-cell SynCAM immunofluorescence comparisons, 3000 dissociated hippocampal cells were plated per well of a 12-well plate. For all other experiments, cells were cultured at a density of 100,000 cells/well of a 12-well plate. For quantitative reverse transcription polymerase chain reaction (qRT-PCR) experiments cells were attached directly to plates coated with poly-L-lysine (Sigma); for all other experiments cells were cultured on glass coverslides coated with poly-L-lysine. Cells were allowed to attach to poly-L-lysine coated substrate in plating media (MEM (Invitrogen), 10% FCS, 20 mM dextrose, 25 units/ml penicillin and 25 μg/ml streptomycin) for 5–6 h. Media was then changed to maintenance media (Neurobasal medium (Invitrogen), 1× B-27 supplement (Invitrogen), 0.5 mM Glutamax (Invitrogen), 50 units/ml penicillin, 50 μg**/**ml streptomycin, and 0.07% β-mercaptoethanol (Sigma)). Half changes of maintenance media were performed every 3–4 days in culture. Primary hippocampal cultures were infected with lentivirus at 1–2 days *in vitro* (DIV). For saturating transduction with lentivirus, 20,000 infectious lentiviral particles (as calculated by our titration method) were added per well of a 12-well plate; for sub-saturating transduction 2000 infectious lentiviral particles were added. For studies using memGFP lentivirus, 100 infectious particles were additionally added. Cells were fixed and stained at 13–15 DIV for imaging experiments; electrophysiology recordings were made with cells at 13–16 DIV.

### Quantitative Western Blotting

Standard sodium dodecyl sulfate polyacrylamide gel electrophoresis (SDS-PAGE) western blotting procedures using nitrocellulose membranes were performed as previously described (Fowler et al., 2016b). Two-color near-infrared blots were imaged with an Odyssey-Fc quantitative western blot system (LI-COR). Primary antibodies and dilutions used were mouse anti-actin 1:2000 (Millipore, clone C4) and rabbit anti-SynCAM1–3 1:1000 (Pierce, PA3-16744); secondary antibodies donkey anti-mouse IRDye 680RD and anti-rabbit IRDye 800CW (LI-COR) were used at 1:1000. Intensities were normalized to actin loading controls. KD efficiency was calculated by comparing levels relative to the Scrambled 1–3 amiRNA control conditions set to 1. The representative blot shown is a composite image made by re-arranging lanes of a single blot image at the same projection intensity.

### qRT-PCR

First-strand cDNAs were synthesized from total RNA isolated from cultured hippocampal neurons using Superscript III reverse transcriptase (Invitrogen) with oligodT primers for 50 min at 50°C. Primer pairs used to measure *cadm* mRNA levels were cadm1_F: 5′-GAAGGACAGCAGGTTTCAGC-3′, cadm1_R: 5′-ACCAGGACTGTGATGGTGGT-3′, cadm2_F: 5′-TCCTGATCGAATGGTTGTGA-3′, cadm2_R: 5′-TGGGATCGTGTACAATGAGG-3′, cadm3_f: 5′-CCTGGAGAAAAGGTGACCAA-3′, cadm3_R: 5′-ATGGTTCACAGAGCACACGA-3′. qRT-PCR was performed using SYBR Green reagents (Kapa Biosystems) using standard parameters on a StepOnePlus Real-Time PCR System (Applied Biosystems). Values and relative expression levels were compared using the ΔΔC_t_ method.

### Immunolabeling

Cells on glass coverslides were fixed with 4% paraformaldehyde and 4% sucrose in PBS for 15 min at 4°C. Cells were then permeabilized for 5 min with 0.25% Triton-X100 in PBS, and blocked for 1 h at RT with blocking solution (1% Roche blocking solution (Roche), 10% BSA (Sigma), 1% normal donkey serum (Jackson ImmunoResearch), and 1% normal goat serum (Jackson ImmunoResearch) in PBS). Cells were then incubated with primary antibodies in blocking solution overnight at 4°C. Primary antibodies and dilutions used were rabbit anti-Synapsin1 1:500 (EMD Millipore, AB1543), mouse anti-PSD-95 1:350 (Neuromab, clone K28/43), chicken anti-GFP 1:2000 (Aves Labs, GFP-1020), and rabbit anti-SynCAM1–3 1:500 (Pierce, PA3-16744). The next day, cells were washed 3× 5 min with PBS and incubated with secondary antibody in blocking solution for 1 h at RT. All secondary antibodies were from Jackson Laboratories and used at 1:500–goat anti-chicken Alexa Fluor 488, donkey anti-mouse or anti-rabbit Cy3, and donkey anti-rabbit Cy5. Cells were washed 3× 5 min with PBS and mounted on slides with Fluoromount G with DAPI (Southern Biotech).

### Microscopic Imaging

For transduction rate experiments, neurons were imaged on a Nikon Eclipse TE300 microscope using a 20× air objective (0.45 NA), Till Photonics monochromator light source, Retiga EXi CCD camera (Q Imaging) and SimplePCI software (Hamamatsu, Inc., Houston, TX, USA). For Figures 3B,C, cells were imaged at 20× (Figure 3B) using an air (0.75 NA) objective or at 60× (Figure 3C) using an oil-immersion objective (1.40 NA) on an Eclipse 80i microscope with a DS-Qi1Mc camera, Intensilight C-HGFI light source and Elements software (Nikon). For Figure 3D, cells were imaged live in aCSF (see “Electrophysiology” Section for recipe) with a 40× water-immersion objective (0.8 NA) using an Eclipse FN1 microscope with a DS-Qi1Mc camera, Intensilight C-HGFI light source and Elements software (Nikon). All other neurons were imaged on an inverted Nikon TU-2000 confocal microscope using EZ-C1 software. For single-cell comparisons of SynCAM1–3 immunofluorescence, images were obtained using a 20× air objective (0.75 NA) and the example images in Figures 4A,B were obtained using a 60× water-immersion objective (1.2 NA). All other images were obtained with a 100× oil-immersion objective (1.45 NA). The presence or absence of nlsGFP was validated by visual comparison of DAPI and GFP staining. For transduction rate comparisons, 20 images of DAPI (350 nm) and corresponding GFP (488 nm) signal were taken at random positions across four coverslides per condition. For single-cell SynCAM1–3 immunofluorescence experiments up to 20 neurons that did not overlap with neighboring cells were selected for imaging for each condition. Eight neurons were imaged for a secondary antibody-only condition for analysis of background fluorescence. Sequential scanning for each channel (488, 543 nm) was performed and the average of three scans was taken at 1024 × 1024 pixel resolution. For all other experiments, pyramidal cells were selected by morphology and cells were imaged if they had 2–3 primary or secondary small diameter dendrites (~1–2 μm not including spines or protrusions) that originated at the soma with no further branches in a single field of view at high magnification, that terminated within a short distance of the soma (usually <100 μm), and that were visually discernable from additional GFP-positive processes and background immunofluorescence. Cells were sampled evenly between isolations. Sequential scanning for each channel (488, 543, 633 nm) was performed and the average of three scans was taken at 2048 × 2048 pixel resolution. For all experiments, images were obtained with constant pinhole, laser intensity, and detector gain settings, and the experimenter was blinded to the conditions.

### Image Analysis

Each color channel was saved independently as grayscale 16-bit TIFF files. For transduction rate comparisons, the number of total cells in an image were determined by manually counting DAPI nuclei; the number of transduced cells was determined by manually counting nlsGFP+ nuclei. For single-cell SynCAM1–3 KD comparisons, binary masks were made of each neuron using Image-Pro 6.3 software (Media Cybernetics) using outlines from 488 nm images. Background debris was cleared from the masks manually using GIMP 2 software (The GIMP Team). SynCAM immunostaining intensity was calculated within the confines of the outline of the neuron defined by the binary masks using a custom program in MATLAB (Mathworks). The average fluorescence intensity for neurons in the secondary antibody only condition was used to measure background signal and was subtracted from the SynCAM staining intensity for each image. For dendrite analysis, using 488 nm images, individual basal dendrite segments averaging ~30 μm in length were selected and binarized manually using Image-Pro 6.3 software (Figures 2A,B). Binary masks were then used in a custom MATLAB program to automatically detect and compare puncta from corresponding 543 nm and 633 nm images (Figure 2B). Briefly, the program calculated the average fluorescent intensity of corresponding images in the binarized GFP region, set a threshold for including pixels in puncta detection (1.5× the mean value for each dendrite), and automatically detected puncta that were >4 contiguous pixels within the confines of the binarized GFP mask. Only puncta >0.15 μm^2^ were considered for density and size analysis. Overlapping pre- and postsynaptic puncta were counted as synapses. Dendrite lengths were measured manually using Image-Pro 6.3 software. Puncta density was calculated by dividing number of puncta by dendrite length. Puncta area was reported by our custom program. Experimenters were blinded to conditions during image analysis.

### Electrophysiology

Whole cell recordings were performed on identified pyramidal neurons in hippocampal cultures using an upright Nikon FN1 microscope with fluorescence imaging capabilities. Recording electrodes (2.8–3.8 MΩ) were filled with an intracellular solution containing (mM): 6 NaCl, 4 NaOH, 130 Cs-gluconate, 11 EGTA, 1 CaCl_2_, 1 MgCl_2_, 10 HEPES, 2 Na_2_ATP, and 0.2 Na_2_GTP. The intracellular solution was pH 7.4 and 296 mOsm. All neurons were studied under voltage clamp conditions with an Axopatch 200A or MultiClamp 700A amplifier (Molecular Devices). Neurons were held at *V*_H_ = −70 mV using pipettes in whole cell patch configuration. Signals were filtered at 3 kHz and sampled at 30 kHz using p-Clamp software (version 10, Molecular Devices). Liquid junction potentials were not corrected. Extracellular solution (aCSF; containing (mM): 125 NaCl, 3 KCl, 1.2 KH_2_PO_4_, 1.2 MgSO_4_, 25 NaHCO_3_, 10 dextrose, and 2 CaCl_2_) was continuously perfused and drugs were bath applied to isolate quantal glutamatergic signaling (TTX, 1 μM and Gabazine, 3 μM).

### mEPSC Analysis

Digitized waveforms of quantal synaptic events were analyzed using MiniAnalysis software (Synaptosoft). Only traces from cells held under voltage clamp with a series resistance <25 MΩ were used for analysis. All events >5 pA were counted for 1–2 min of trace ~5 min after application of drug. All events were used to calculate frequency values. The average mEPSC projection of all discrete events for each neuron was used for peak analysis measurements by automated fitting of amplitudes and decay kinetics (single exponential, 90–10%) with MiniAnalysis software. Noisy, misaligned and non-discrete events were manually removed prior to peak analysis. The experimenters were blinded to the conditions for recording and trace analysis.

### Statistics

Normality of data was determined by Shapiro-Wilk tests in R (R Foundation for Statistical Computing). *P* values obtained by statistical comparisons of two sample groups used Student’s two-tailed, unpaired *t*-tests in Microsoft Excel and comparisons of more than two sample groups used one-way ANOVAs followed by Tukey’s *post hoc* pairwise comparisons in R.

## Results

### Single and Multi-Gene Knockdown Lentiviral Constructs

**Figure 1:**
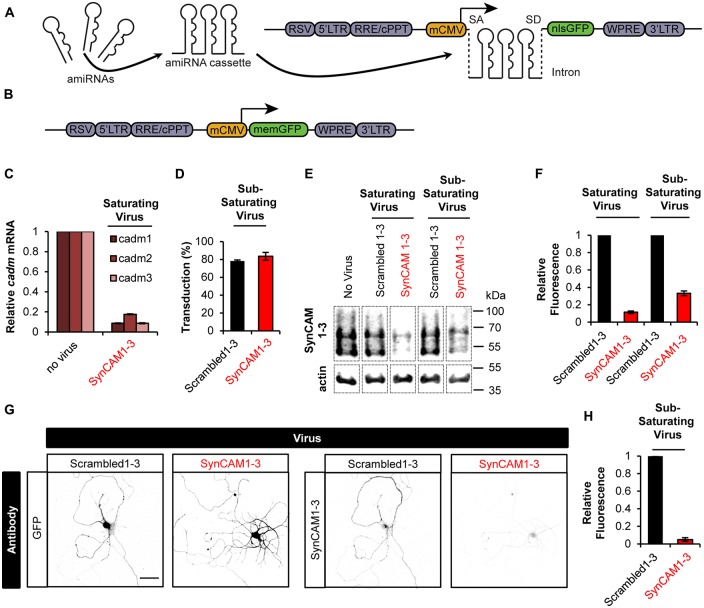
**Sub-saturating lentiviral artificial microRNA (amiRNA) transduction potently knocks down synaptic cell adhesion molecules 1–3 (SynCAM1–3) in amiRNA+ cells while keeping a subpopulation of wild type (WT) amiRNA- cells. (A)** Lentiviral knockdown (KD) vectors were generated with a minimal CMV (mCMV) promoter, intronic amiRNAs, and nlsGFP. RSV, Rous sarcoma virus promoter; LTR, long-terminal repeat; RRE, Rev-response element; cPPT, central polypurine tract; WPRE, woodchuck hepatitis virus posttranscriptional regulatory element; SA, splice acceptor; SD, splice donor. **(B)** memGFP-expressing lentiviral vector for dendrite labeling. **(C)** Comparison of *cadm1–3* mRNA levels at 14–15 days *in vitro* (DIV) by quantitative reverse transcription polymerase chain reaction (qRT-PCR) in cultured rat hippocampal neurons using saturating viral titers relative to levels in uninfected control cultures. *n* = 2 experiments. **(D)** Transduction rate of cultured neurons at 14–15 DIV using sub-saturating viral titers. *n* = 2 experiments. **(E)** Representative blots and **(F)** relative SynCAM1–3 protein levels at 13–15 DIV measured by quantitative western blotting of cultured neurons following indicated amiRNA lentiviral treatments at saturating or sub-saturating viral titers compared to Scrambled1–3 amiRNA control treatments. Actin was used as a loading control. *n* = 4 (saturating) or *n* = 2 (sub-saturating) experiments. A single gel image of the same intensity was cropped as marked by dotted lines and rearranged for presentation. **(G)** Representative images and **(H)** quantification of relative SynCAM1–3 immunofluorescence at 14 DIV of individual neurons cultured at low density normalized to immunofluorescent intensity of control Scrambled 1–3 amiRNA treated neurons. Scale, 50 μm. Scrambled1–3 condition *n* = 13 cells/2 coverslips, SynCAM1–3 condition *n* = 20 cells/2 coverslips. Error bars, SEM.

Single and multi-gene KD was achieved through the use of inhibitory RNA (RNAi) targeting sequences in an enhanced amiRNA backbone either singly or chained in a mobile cassette (Fowler et al., 2016b). We combined amiRNAs targeting rat SynCAM1–3 in single, double and triple-gene KD combinations, as well as three scrambled amiRNA sequences targeting no known genes (Scrambled1–3), into a lentiviral destination vector with a nuclear localized GFP (nlsGFP) reporter (Fowler et al., 2016a; Figure 1A). These amiRNAs were expressed from an intron to prevent degradation of the mRNA to allow robust reporter expression (Chung et al., 2006). We also generated a separate lentiviral vector driving expression of a plasma membrane-localized GFP (memGFP) reporter used to label dendrites and spines (Figure 1B). We previously characterized these chained amiRNAs targeting SynCAM1–3, which are encoded by the cell adhesion molecule 1–3 (*cadm1–3*) genes, as highly efficient for KD of SynCAM1–3 in rat primary hippocampal cultures at 13–15 DIV by western blot (Fowler et al., 2016b). For further validation, we performed qRT-PCR for *cadm1–3* mRNA levels in hippocampal cultures following transduction with saturating levels of SynCAM1–3 amiRNAs. Results showed >90% KD of *cadm1* and *3*, and >80% KD of *cadm2* mRNAs, confirming that our amiRNAs are effective for KD of all three SynCAMs (Figure 1C).

Typically, Lentiviral RNAi experiments are performed at saturating transduction levels to ensure the highest level of KD. However, saturating amiRNA virus prevents a population of non-transduced cells retaining WT SynCAM1–3 expression levels. Because we eventually wanted to compare synapse development in both WT and SynCAM1–3 KD neurons on the same coverslip, we infected cultures with sub-saturating amounts of SynCAM1–3 amiRNA or control Scrambled1–3 amiRNA virus. We counted nlsGFP+ DAPI-stained nuclei and saw that ~80% of cells were transduced in both cases (Figure 1D), ensuring that ~20% of cells retained WT SynCAM expression levels.

Because our previous characterizations of SynCAM1–3 KD by western blot (Fowler et al., 2016b) and in the current study with qPCR (Figure 1C) used saturating viral levels, we wanted to confirm that sub-saturating transduction still produced robust KD. Using quantitative western blotting with an antibody that recognizes SynCAM1–3 (Biederer et al., 2002), but not SynCAM4 (Fogel et al., 2007), we showed ~90% KD with saturating and ~70% KD with sub-saturating virus (Figures 1E,F). Because around 20% of cells retained WT SynCAM1–3 expression in sub-saturating virus, we reasoned that the observed 70% KD likely underrepresented the actual KD amount in SynCAM1–3 amiRNA+ cells. Therefore, we cultured cells at very low densities to allow imaging of individual cells transduced with sub-saturating concentrations of SynCAM1–3 or Scrambled1–3 amiRNA lentivirus. As measured by immunofluorescence intensity for SynCAM1–3 antibody, SynCAM1–3 amiRNA+ cells had ~95% KD compared to Scrambled1–3 amiRNA+ cells (Figures 1G,H). This confirmed that sub-saturating concentrations of the amiRNA lentivirus effectively eliminated targeted protein expression in individual transduced neurons, while maintaining a subpopulation of amiRNA− cells to be used as in-culture WT controls.

### SynCAM1–3 Redundantly Set Synapse Density and Size During Synaptogenesis

**Figure 2:**
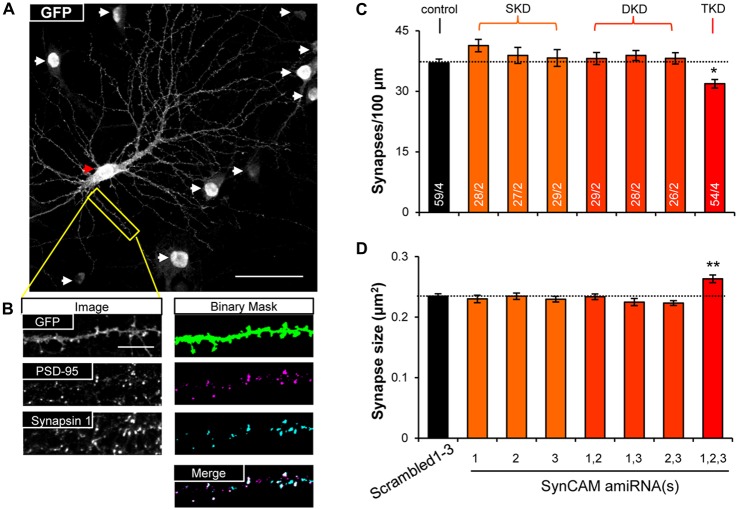
**SynCAM1–3 function redundantly to set synapse density and size. (A)** Representative 60× confocal microscopy image of cultured rat hippocampal neurons at 14 DIV treated with sub-saturating lentivirus carrying Scrambled1–3 amiRNAs linked to nlsGFP, and very low titer memGFP lentivirus. An individual pyramidal cell co-labeled by memGFP and nlsGFP is marked by a red arrowhead. White arrowheads mark nuclei in the field of view only expressing nlsGFP. Scale, 50 μm. **(B)** Sample images of automated puncta and synapse detection from co-immunostaining for presynaptic protein Synapsin 1 and postsynaptic protein PSD-95 following manual selection of an individual dendrite segment from the image in **(A)**. Synapses are defined as the co-localized regions of pre- and postsynaptic puncta (white areas in merged image). Scale, 10 μm. **(C)** Average synapse density and **(D)** average synapse puncta area from 13 to 15 DIV neurons co-transduced with memGFP and sub-saturating amounts of the SynCAM amiRNAs listed or control Scrambled1–3 amiRNAs. *n* = cells/isolations is listed on the bars. SKD, single knockdown; DKD, double knockdown; TKD, triple knockdown. *p < 0.05, **p < 0.01, one-way analyses of variance (ANOVA) with Tukey’s *post hoc* pairwise comparisons on cell average values. Error bars, SEM.

To test functional redundancy of SynCAM1–3 during synapse formation, we infected hippocampal cultures at sub-saturating amounts with all possible combinations of lentivirus carrying single, double, and triple amiRNAs against SynCAM1–3, as well as the Scrambled1–3 amiRNA control. We also infected a small subset of neurons (~1%) with memGFP lentivirus to enable imaging of dendrites. To detect differences in synapse formation, we immunolabeled cultures at 13–15 DIV with antibodies to GFP, the postsynaptic protein PSD-95 and the presynaptic protein Synapsin 1 and imaged nlsGFP/amiRNA+ cells that were also labeled with memGFP using confocal microscopy (Figure 2A). Following manual selection of dendrites on pyramidal cells, we used automated detection of PSD-95 and Synapsin 1 puncta. We defined synapses as regions of co-localization of these pre- and postsynaptic puncta along dendrites (Figure 2B). Only SynCAM1–3 KD, but not single or double KD, reduced synapse density by nearly 20% (Figure 2C) and increased the area of co-localization by ~10% (Figure 2D), demonstrating that SynCAM1–3 function redundantly to set synapse number and size in developing neurons.

### Mosaic Knockdown Reveals SynCAM1–3 Set Synapse Density and Size Through Postsynaptic Mechanisms

**Figure 3:**
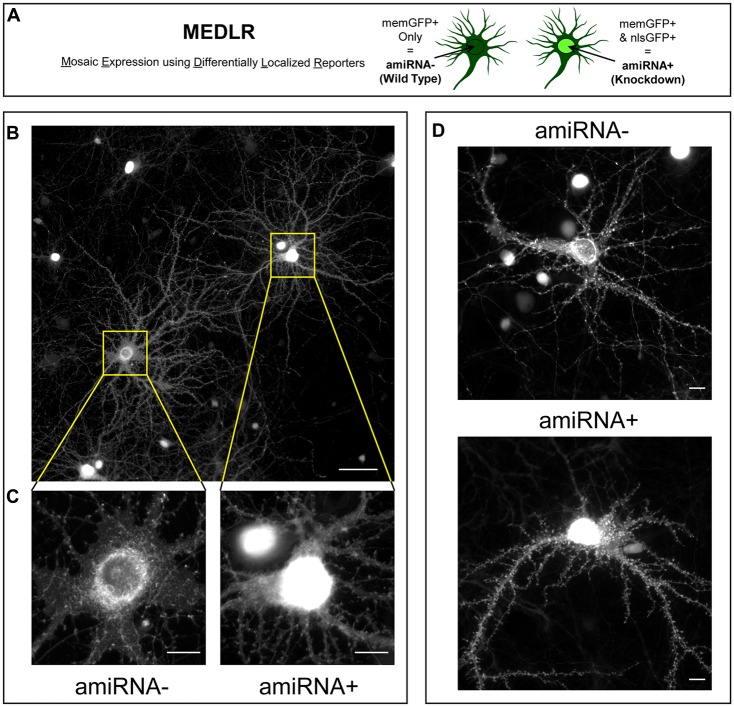
**Mosaic Expression using Differentially Localized Reporters (MEDLR) is a novel method to compare WT and KD neurons on the same coverslip. (A)** MEDLR uses combinatorial lentiviral transgenesis with memGFP lentivirus and nlsGFP-linked amiRNA lentivirus to compare WT and KD cells. **(B)** Representative immunolabled GFP images at 20× or **(C)** 60× magnification of memGFP-labled amiRNA− and amiRNA+ neurons at 15 DIV in cultures transduced with sub-saturating amounts of Scrambled1-3 amiRNAs. **(D)** Representative 40× images of live GFP fluorescence in 15 DIV amiRNA− and amiRNA+ neurons. **(B–D)** Note the perisynaptic memGFP accumulation that could potentially be misinterpreted as nlsGFP in amiRNA− cells. **(B)** Scale, 50 μm; **(C,D)** Scale, 10 μm.

We sought to determine if the phenotypes due to SynCAM1–3 KD are caused by pre- or postsynaptic loss. Sub-saturating infection with amiRNA lentivirus results in non-transduced cells retaining WT SynCAM1–3 expression levels. Combinatorial application of memGFP lentivirus enables labeling of these WT cells for imaging, which is not possible when an RNAi payload is directly linked to the neurite-labeling fluorophore. Therefore this new methodology, named Mosaic Expression using Differentially Localized Reporters (MEDLR), allows direct comparisons of WT (memGFP+ only) and KD (memGFP+/nlsGFP+) neurons on the same coverslip (Figure 3A). Using MEDLR, amiRNA− WT cells were readily distinguished from amiRNA+ KD cells when visually scanned for the presence or absence of nlsGFP fluorescence in memGFP-labeled fixed cell preparations under low magnification (Figure 3B). The distinction between amiRNA− and amiRNA+ cells was even more apparent at higher magnifications (Figure 3C). Further, MEDLR was used to differentiate amiRNA− and amiRNA+ cells in live cultures (Figure 3D), showing that this methodology is suitable for approaches such as live cell imaging and electrophysiology.

Crucially, for proteins located on both axons and dendrites, such as SynCAM1–3, MEDLR allows the discrimination between pre- and postsynaptic sites of action. This is because in a culture infected with sub-saturating SynCAM1–3 amiRNAs, the majority of axons (~80%) available to form connections with WT neurons have ~5% of wildtype levels of SynCAM1–3. Therefore if presynaptic SynCAM1–3 is necessary for proper synapse formation then WT cells would be expected to display a similar phenotype to SynCAM1–3 KD neurons. However, if postsynaptic SynCAM1–3 instructs synapse development, no phenotype would be expected in WT cells. We note that as used here, MEDLR cannot determine if postsynaptic SynCAM1–3 is necessary for synapse formation, because in amiRNA+ cells both pre- and postsynaptic SynCAM1–3 would be removed. Instead, MEDLR can determine if the presence of postsynaptic SynCAM1–3 is sufficient for synapse formation.

**Figure 4:**
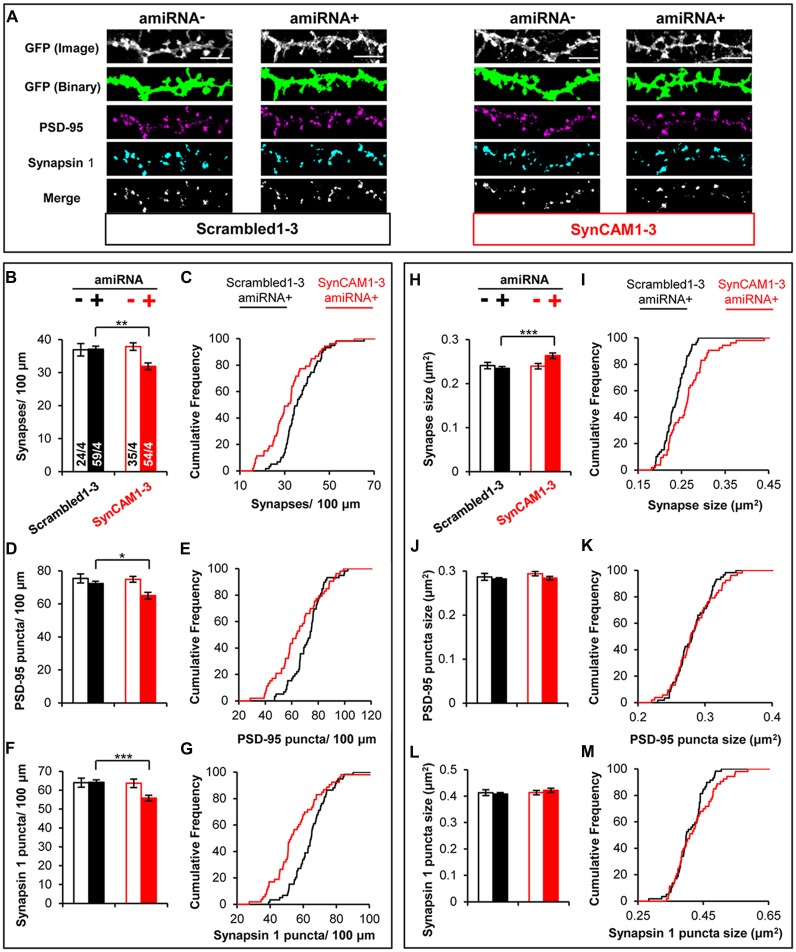
**MEDLR shows synaptic phenotypes are due to postsynaptic SynCAM1–3 depletion. (A)** Representative images of memGFP-labled basal dendrites from amiRNA− and amiRNA+ neurons at 15 DIV in cultures transduced with sub-saturating amounts of Scrambled1–3 or SynCAM1–3 amiRNAs. Automated puncta detection for PSD-95 and Synapsin 1 immunostaining and synapses (merge) was performed after manual selection of GFP masks. Scale, 5 μm. **(B,D,F)** Average dendritic puncta density as indicated on graph of amiRNA− and amiRNA+ neurons and **(C,E,G)** corresponding cumulative puncta density distribution plot (%) of amiRNA+ neurons from cultures transduced with Scrambled1–3 or SynCAM1–3 amiRNAs. **(H,J,L)** Average puncta area as indicated on graph of amiRNA− and amiRNA+ neurons and **(I,K,M)** corresponding cumulative puncta area distribution plot (%) of amiRNA+ neurons from cultures transduced with Scrambled1–3 or SynCAM1–3 amiRNAs. *n* = number of cells/isolations as indicated on bars from 13 to 15 DIV cultures. *p < 0.05, **p < 0.01, ***p < 0.001, *t* test on cell average values. Cumulative distribution plots represent cell average values. Error bars, SEM.

We used MEDLR to compare synaptic phenotypes between amiRNA− and amiRNA+ cells in cultures transduced with sub-saturating amounts of Scrambled1–3 or SynCAM1–3 amiRNA lentivirus (Figure 4A). WT amiRNA− cells did not display reduced synaptic density compared to Scrambled1–3 amiRNA+ neurons (Figures 4B,C) suggesting that the reduction in SynCAM1–3 amiRNA+ neurons was due to depletion of postsynaptic SynCAM1–3. Further, analysis of PSD-95 (Figures 4D,E) and Synapsin 1 puncta density (Figures 4F,G) showed that both were reduced along dendrites of SynCAM1–3 amiRNA+ neurons but not on Scrambled1–3 amiRNA+ neurons or amiRNA− cells, positing that reduced synapse density is due to the inability to recruit both pre- and postsynaptic structures. MEDLR also revealed no difference in synapse area in amiRNA− cells compared to Scrambled1–3 amiRNA+ neurons (Figures 4H,I), again suggesting that the increased synapse size is because of postsynaptic SynCAM1–3 loss. In contrast to puncta density, there was no difference in mean PSD-95 (Figure 4J) or Synapsin 1 (Figure 4L) puncta size between Scrambled1–3 and SynCAM1–3 amiRNA− or amiRNA+ cells, although we note that distribution plots indicated a subset of amiRNA+ cells that had larger mean PSD-95 and Synapsin 1 puncta areas (Figures 4K,M). Given that roughly twice as many Synapsin 1 and PSD-95 structures are detected than synapses, we cannot rule out that subtle increases in the size of Synapsin 1 and PSD-95 puncta size in SynCAM1–3 KD cells may contribute to the observed, enlarged synapse size. Alternatively, the increased synapse area may be due to the increased overlap of a subset of pre- and postsynaptic markers, which would be indicative of an effect primarily at trans-synaptic complexes. Further, it should be noted that increased puncta overlap in SynCAM1–3 amiRNA+ cells is unlikely to be from a non-specific effect that generally brings puncta in closer apposition, because individual puncta density was also decreased in these cells (Figures 4D–G).

### SynCAM1–3 Knockdown Affects Quantal Transmission Through a Postsynaptic Mechanism

**Figure 5:**
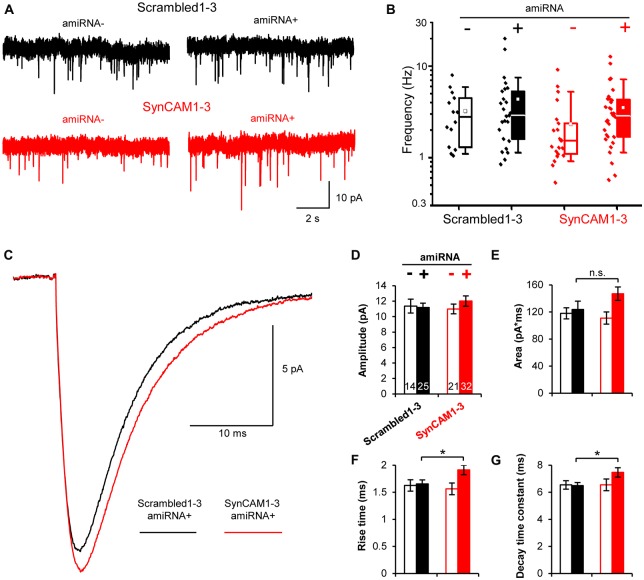
**Postsynaptic SynCAM1–3 loss does not alter miniature excitatory postsynaptic current (mEPSC) frequency but broadens mEPSC events. (A)** Representative whole-cell patch clamp mEPSC traces of amiRNA− and amiRNA+ neurons from cultures treated with sub-saturating amounts of Scrambled1–3 or SynCAM1–3 amiRNAs. **(B)** Box and whisker plot of mEPSC frequencies with individual data points plotted to left of boxes. Box = 25–75%, whiskers = 10–90%, line = median, square = mean. **(C)** Average mEPSC event traces for amiRNA+ cells in cultures transduced with Scrambled1–3 or SynCAM1–3 amiRNAs. **(D–G)** Average mEPSC measurements as indicated on graph for amiRNA− and amiRNA+ cells in cultures transduced with Scrambled1–3 or SynCAM1–3 amiRNAs. **(B–G)** *n* = number of cells as indicated on bars in **(D)** from three isolations recorded at 13–16 DIV. *p < 0.05, n.s., not significant, *t* test between Scrambled1–3 and SynCAM1–3 amiRNA+ conditions using cell average values. Error bars, SEM.

The MEDLR approach enabled us to determine the extent to which quantal glutamatergic synaptic transmission was altered in SynCAM1–3 KD neurons. Using whole-cell patch clamp electrophysiology, we measured mEPSCs at 13–16 DIV onto amiRNA− and amiRNA+ neurons transduced with sub-saturating amounts of Scrambled1–3 or SynCAM1–3 amiRNAs (Figure 5A). Distributions of average mEPSC frequencies across cells were highly variable, logarithmically distributed, and spanned nearly two orders of magnitude (Figure 5B). Because of the spread of frequencies, we did not observe a systematic difference between any group suggesting that SynCAM1–3 do not significantly impact the net frequency of quantal release. This result highlights that quantal release frequency is a product of multiple factors, including intrinsic release probability (Branco and Staras, 2009), in addition to synapse density, and is not necessarily an accurate measure of synapse number.

We also compared mEPSC event traces between groups. On average, mEPSCs were larger in SynCAM1–3 KD neurons compared to Scrambled1–3 amiRNA+ cells (Figure 5C). Waveform fitting analysis showed that while peak amplitude was not different between Scrambled1–3 and SynCAM1–3 amiRNA+ cells (Figure 5D; *p* = 0.36, *t* test), peaks in SynCAM1–3 KD neurons tended to have a larger area (Figure 5E; *p* = 0.14, *t* test) and had significantly longer rise times and decay-time constants (Figures 5F,G); indicating that SynCAM1–3 KD causes broader mEPSCs. MEDLR showed no differences between amiRNA− cells and Scrambled1–3 amiRNA+ neurons, demonstrating that postsynaptic depletion of SynCAM1–3 enlarges mEPSCs (Figures 5D–G). Additionally, there was no difference in average cell capacitance between conditions (Scrambled amiRNA+ = 52.5 ± 3.2 pF, *n* = 25; Scrambled amiRNA− = 55.3 ± 3.5 pF, *n* = 14; SynCAM1–3 amiRNA+ = 55.8 ± 3.4 pF, *n* = 32; SynCAM1–3 amiRNA− = 54.0 ± 4.8 pF, *n* = 20), indicating that changes in mESPC kinetics was not due to a change in cell size. Collectively, these results are consistent with an increased synapse size caused by postsynaptic loss of SynCAM1–3, and lend further evidence that defining synapses as juxtaposed pre- and postsynaptic puncta accurately represents functional contacts.

## Discussion

This report describes the first investigation into the functional redundancy of SynCAM1–3 during excitatory synapse development. We present three main observations that enhance our understanding of SynCAM synaptogenic functions: (1) only triple KD of SynCAM1–3 reduced synapse number, implying functional compensation during synapse formation; (2) triple KD increased synapse and mEPSC size, suggesting that intact SynCAM1–3 signaling functions redundantly to limit the physical size of trans-synaptic complexes; and (3) use of MEDLR provides strong evidence that postsynaptic, not presynaptic, SynCAM1–3 regulate synapse density and size. Additionally, the development of MEDLR should prove useful for future investigations not only in neuronal cultures, but in other adaptations where comparisons of WT cells to treated cells in the same culture is beneficial.

The observation of SynCAM1–3 functional redundancy appears to conflict with previous reports suggesting SynCAM1 knockout (KO) alone reduces synapse density in excitatory neurons (Robbins et al., 2010; Cheadle and Biederer, 2012; Giza et al., 2013; Park et al., 2016). While we cannot rule out the possibility that residual SynCAM1, due to incomplete KD, is sufficient for correct synaptogenesis, this seems unlikely because of high SynCAM1 KD potency using our enhanced amiRNA. Further, this would not explain why the triple KD decreased synapse number.

One possibility for this discrepancy could be global KO vs. mosaic KD, however, this does not seem likely considering ~80% of cells were transduced with amiRNAs in our experiments. Moreover, for the synaptogenic adhesion molecule neuroligin-1, global KO does not alter synapse density, whereas sparse KD does (Kwon et al., 2012); this is the opposite of what is observed for SynCAM1 where global KO reduced synapse density (Robbins et al., 2010), whereas sparse KD using transfection of SynCAM1 shRNAs does not (Burton et al., 2012 and unpublished observations). Further, Kwon et al. (2012) demonstrated that at 1:1 mix of neuroligin-1 WT and KO neurons (50% of cells KO) in culture strongly reduced synapse formation on KO neurons, whereas our MEDLR experiments (80% of cells KD) do not show a phenotype from SynCAM1 KD alone. Together, this argues against global-vs.-local SynCAM1 depletion underlying differences in synapse formation. Unfortunately, due to a technical limitation of combinatorial viral transfection we were not able to investigate synapse development in cultures transduced sparsely with amiRNA virus because insufficient cells were co-transduced with both memGFP and amiRNAs.

Alternatively, differences in analysis methods may complicate direct comparisons. For instance, the discrepancy may stem from the different cell types assayed. SynCAM1 KO mice showed an overall reduction of hippocampal excitatory synapse number as measured by electron microscopy of whole tissue (Robbins et al., 2010). However, this technique does not differentiate between synapses onto interneurons or pyramidal cells. Indeed, a later study showed a specific reduction of excitatory inputs on hippocampal interneurons due to SynCAM1 KO (Park et al., 2016). Since the current study investigated pyramidal cells, it is possible that cell type differences account for the conflicting observations. Additionally, other reports have used dendritic spine density and mEPSC frequency as indirect measurements of synapse number (Robbins et al., 2010; Giza et al., 2013; Park et al., 2016), but these are by nature correlative measurements. Importantly, the only other SynCAM1 loss-of-function study to our knowledge that investigated synapse density used RNAi-mediated KD in hippocampal cultures also found no change in synapse number as measured by Synapsin puncta along dendrites (Burton et al., 2012). Taken together with our studies, it is probable that a more stringent measurement of synapse density by immunolabeling is not altered by SynCAM1 depletion alone.

We also present imaging and electrophysiological data suggesting that triple KD of SynCAM1–3 increased synapse size. It is interesting to note that our imaging results showed enlarged synapses as measured by overlapping pre- and postsynaptic puncta area, yet the mean value of individual puncta was unchanged (Figures 4H–M). We believe this is indicative of changes primarily at trans-synaptic complexes for the following reasons: (1) synapses were smaller compared to individual puncta sizes so mean values for individual puncta would be less affected by similar changes in size; (2) the distribution data suggested a tendency towards increased individual puncta size in some cells; and (3) there were more individual puncta than synapses, with the possibility that the non-synaptically associated fraction is not enlarged. The combination of these factors could easily prevent a mean size shift for individual puncta size.

Moreover, results from previous studies support a model where intact SynCAM1–3 signaling limits synapse size at trans-synaptic structures. SynCAM1’s ability to bind in *trans* is dependent on its ability to form oligomers in *cis* (Fogel et al., 2011). Intriguingly, postsynaptic disruption of SynCAM1 *trans* interactions by *cis*-binding to a dominant-negative SynCAM1 extracellular domain similarly increased immunolabeled synapse size (Fogel et al., 2011). SynCAM heteromers also form in *cis* to promote *trans* binding (Frei et al., 2014); therefore it is reasonable to infer that the dominant-negative SynCAM1 extracellular region disrupted trans-synaptic adhesion of all SynCAM1–3, leading to increased synapse size. Together with the recent report that postsynaptic SynCAM1 localizes to and shapes the synaptic periphery (Perez de Arce et al., 2015), it is tempting to speculate that postsynaptic SynCAM1–3 limit synapse size through a mechanism involving the development of the synaptic edge.

Further, the results using MEDLR to compare WT and KD neurons in the same cultures suggest that postsynaptic SynCAM1–3 instruct synapse formation. Because SynCAMs are assumed to function homo- or heterophilically in *trans* across the synapse, removal of SynCAMs from either the pre- or postsynaptic side would be expected to impair synapse development. Yet our observations show that postsynaptic expression of SynCAM1–3 in WT cells was sufficient for correct synapse development, even when the majority of presynaptic SynCAM1–3 was removed. These results imply that postsynaptic, and not presynaptic, SynCAM1–3 are the major determinant of SynCAM-mediated synapse formation, and posits a previously unrecognized postsynaptic function for SynCAM1–3. This was surprising due to the current assumption in the field that presynaptic SynCAMs dictate synapse formation since presentation of ectopic SynCAMs induces presynaptic, but not postsynaptic structures (Breillat et al., 2007; Czöndör et al., 2013). The overall reduction of Synapsin 1 and PSD-95 puncta upon SynCAM1–3 KD, however, indicates postsynaptic SynCAM1–3 are responsible for recruiting and/or stabilizing *both* pre- and postsynaptic structures. This notion is supported by the observation that postsynaptic SynCAM1 coordinates the assembly of both pre- and postsynaptic complexes through Farp1 (Cheadle and Biederer, 2012). However, Farp1 exclusively binds SynCAM1 and is unlikely to mediate the redundancy of SynCAM2 and 3.

Considered together, these results raise the question: how can postsynaptic SynCAM1–3 guide synapse assembly of both pre- and postsynapses when: (1) there is a severely reduced background of presynaptic SynCAM1–3; and (2) SynCAMs themselves lack the ability to induce postsynapse formation? We offer three possible explanations to reconcile these seemingly conflicting observations. First, it is possible that postsynaptic SynCAM1–3 bind to presynaptic SynCAM1–3 on WT axons, forming a much larger number of synapses on these processes to achieve an overall correct density. Second, it is possible that postsynaptic SynCAM1–3 binds residual SynCAM1–3 on KD axons, and this still allows synapse formation. This possibility would necessitate that a relatively tiny amount of presynaptic SynCAM1–3 is sufficient for correct synaptogenesis, whereas postsynaptic SynCAM1–3 removal is more apt to produce a phenotype. Both explanations would still require trans-synaptic SynCAM interactions to assemble a presynapse, which would recruit additional factors to in turn act across the cleft to induce postsynapse development. For example, the release of glutamate at nascent presynapses could trigger postsynapse assembly (Kwon and Sabatini, 2011), because clustering of presynaptic SynCAMs robustly induces functional presynaptic terminals (Biederer et al., 2002; Sara et al., 2005; Hoy et al., 2009; Czöndör et al., 2013).

A third possibility posits that presynaptic SynCAM1–3 are not actually necessary for synapse development. Instead, postsynaptic SynCAM1–3 could bind in *trans* to an unknown presynaptic adhesion molecule to induce presynaptic differentiation. This in turn could also induce postsynapse formation by recruiting additional postsynaptic factors, or may activate postsynaptic SynCAM1–3 to trigger development in a way that clustering in *trans* by SynCAMs does not. On the other hand, SynCAM1–3 could bind in *cis* to synaptogenic factors to both initiate postsynapse formation and signal retrogradely to instruct presynapse assembly. Because SynCAMs associate both in *cis* and *trans* with numerous adhesion molecules such as nectins (Mori et al., 2014), CRTAM (Arase et al., 2005; Boles et al., 2005; Galibert et al., 2005), and integrins (Mizutani et al., 2011; Sugiyama et al., 2013) and modulates receptor tyrosine kinase signaling in *cis* (Kawano et al., 2009; Kim et al., 2011; Sandau et al., 2011; Yamada et al., 2013), these may be worthwhile avenues for future investigations of how postsynaptic SynCAM1–3 guide synapse formation.

## Author Contributions

DKF, JHP and PW conceived and designed the experiments, DKF, JHP and CW performed the experiments and DKF and CW analyzed the data. All authors were involved in drafting the manuscript and provided final approval for submission.

## Funding

This work was supported by National Institutes of Health (NIH) grants from National Institute of Neurological Disorders and Stroke (award no. R01 NS065795) to PW, National Institute of Diabetes and Digestive and Kidney Diseases (award no. R01 DK092651) to JHP and Eunice Kennedy Shriver National Institute of Child Health and Human Development (award no. T32 HD07348) to DKF.

## Conflict of Interest Statement

The authors declare that the research was conducted in the absence of any commercial or financial relationships that could be construed as a potential conflict of interest.
